# Proteomic Analysis of Liver in Rats Chronically Exposed to Fluoride

**DOI:** 10.1371/journal.pone.0075343

**Published:** 2013-09-17

**Authors:** Heloísa Aparecida Barbosa da Silva Pereira, Aline de Lima Leite, Senda Charone, Janete Gualiume Vaz Madureira Lobo, Tania Mary Cestari, Camila Peres-Buzalaf, Marília Afonso Rabelo Buzalaf

**Affiliations:** Department of Biological Sciences, Bauru Dental School, University of São Paulo, Bauru, São Paulo, Brazil; University of Navarra School of Medicine and Center for Applied Medical Research (CIMA), Spain

## Abstract

Fluoride (F) is a potent anti-cariogenic element, but when ingestion is excessive, systemic toxicity may be observed. This can occur as acute or chronic responses, depending on both the amount of F and the time of exposure. The present study identified the profile of protein expression possibly associated with F-induced chronic hepatotoxicity. Weanling male *Wistar* rats (three-weeks old) were divided into three groups and treated with drinking water containing 0, 5 or 50 mg/L F for 60 days (n=6/group). At this time point, serum and livers were collected for F analysis, which was done using the ion-sensitive electrode, after hexamethyldisiloxane-facilitated diffusion. Livers were also submitted to histological and proteomic analyses (2D-PAGE followed by LC-MS/MS). Western blotting was done for confirmation of the proteomic data A dose-response was observed in serum F levels. In the livers, F levels were significantly increased in the 50 mg/L F group compared to groups treated with 0 and 5 mg/L F. Liver morphometric analysis did not reveal alterations in the cellular structures and lipid droplets were present in all groups. Proteomic quantitative intensity analysis detected 33, 44, and 29 spots differentially expressed in the comparisons between control *vs.* 5 mg/L F, control *vs.* 50 mg/L F, and 5 mg/L *vs.* 50 mg/L F, respectively. From these, 92 proteins were successfully identified. In addition, 18, 1, and 5 protein spots were shown to be exclusive in control, 5, and 50 mg/L F, respectively. Most of proteins were related to metabolic process and pronounced alterations were seen for the high-F level group. In F-treated rats, changes in the apolipoprotein E (ApoE) and GRP-78 expression may account for the F-induced toxicity in the liver. This can contribute to understanding the molecular mechanisms underlying hepatoxicity induced by F, by indicating key-proteins that should be better addressed in future studies.

## Introduction

Fluoride (F) has a known therapeutic action against dental caries [1,2] supporting its implementation in public drinking water and dental products for broad use. However, any element when ingested in excessive doses can lead to side effects. In this context, the presence of high levels of F in the water resources results in endemic fluorosis in humans. In animal models, the chronic treatment with high doses of F was shown to affect several tissues. Many proteins and enzymatic systems have been shown to suffer changes upon exposure to high F levels [3,4]. After being absorbed by the gastric-intestinal system, F is distributed to all soft and mineralized tissue through the blood [5].

Many reports have shown that F can cause progressive degeneration of the structure and functions of the skeletal muscles, brain, and spine [6]. In addition, it increases the aerobic metabolism and promotes alterations in the metabolism of cellular free radicals in several organs such as liver, kidneys, and heart [7]. Whitford [5] reported that F is also a potent inhibitor of many enzymes. Several phosphatases are F sensitive in different ranges of degrees, including inorganic pyrophosphatase, acid phosphatase of bone cells, and osteoclastic tartrate-resistant acid phosphatase, inhibited at 3.8, 0.95-3.8 and 3.8-19 mg/L, respectively [8,9,10,11]. Moreover, F at micromolar concentrations induces apoptosis and regulation of the immune response [12,13,14].

Liver represents the main detoxifying tissue by processing, neutralizing, and eliminating toxins from the digestive tract through hepatocyte-mediated enzymatic detoxification systems [15]. Despite many studies in the literature focused on F-based toxicity, the molecular mechanisms underlying the effects on protein expression of chronic F-induced damage remain unclear. Systematic analysis in protein expression after exposure to F may help to identify new toxicity biomarkers in the liver. To address that, liver proteomic analysis was used as a tool allowing for the identification of a large number of proteins simultaneously in rats chronically receiving both low and high levels of F.

## Materials and Methods

### Animals and treatment

Weanling male *Wistar* rats (three-weeks old) were randomly distributed into three groups containing six animals each. The animals were housed in groups of three animals per cage receiving low-F food (AIN-93, 0.69 µgF/g) and water *ad libitum*. This diet presents higher carbohydrate and calorie values than regular diets [16]. The temperature and humidity in the climate-controlled room, which had a 12 h light/dark cycle, were 23±1°C and 40%–80%, respectively. Experimental groups were treated with F (as NaF) at concentrations of 5 or 50 mg/L in drinking water for 60 days. They lead to plasma fluoride levels typically found in humans drinking water containing 1 and 10 mg/L F in human, respectively [17]. The control group received deionized water (vehicle) for the same period. At the end of the study, the animals seemed to be healthy. They were then anaesthetized with ketamine and xylazine and their blood and livers were collected. The right and left lobes were used for histopathological examination and fluoride analysis, respectively. The median lobe was utilized for the proteome and immunoblot analysis. F quantitation was also done in serum. All experimental protocols were approved by the Ethics Committee for Animal Experiments of Bauru Dental School, University of São Paulo (Protocol 001/2009).

### Histopathological Analysis

The right lobe (n=6 per group) was fixed in 10% neutral-buffered formalin, embedded in paraffin, cut into 5 µm sections, and stained with hematoxylin and eosin (HE) using a standard technique. The sections were examined under light microscope (Carl Zeiss, German) and photomicrographs were taken.

### F Quantitation

The left lobes of livers were homogenized in deionized water for 2 min (Marconi, Model MA 102). Serum and liver F concentrations were determined in duplicate after overnight hexamethyldisiloxane (HMDS)-facilitated diffusion [5,18] using the ion-specific electrode (Orion Research, Model 9409) and a miniature calomel electrode (Accumet, #13-620-79) both coupled to a potentiometer (Orion Research, Model EA 940) (n=6 per group). F standards (0.00475 to 0.19 µg F) were prepared in triplicate and diffused in the same manner as the samples. In addition, non diffused standards were prepared to have exactly the same F concentrations as the diffused standards. Comparison of the mV readings demonstrated that the F in the diffused standards had been completely trapped and analyzed (recovery >95%). The mV potentials were converted to µg F using a standard curve with a coefficient correlation of r≥ 0.99.

### Sample Preparation for 2DE

The livers were washed in an ice-cold buffer (100 mM Tris, 1 mM EDTA, 1 mM phenylmethylsulfonyl fluoride (PMSF), pH 7.4), and rapidly frozen in liquid nitrogen. The frozen tissue was homogenized in a cryogenic, model 6770 Freezer Mill (Spex, Metuchen, NJ, USA). For the protein extraction, liver homogenate was incubated in a lysis buffer containing 7 M urea, 2 M thiourea, 4% CHAPS, 1% IPG buffer pH 3-10, 40 mM DTT supplemented with protease inhibitor cocktail (Roche Diagnostics, Mannheim, Germany; 5 µL/mg of tissue) for 1 h at 4°C with occasional shaking. After this period, the homogenate was centrifuged at 15,000 rpm for 30 min at 4°C, and the supernatant containing soluble proteins was recovered [19] ). The proteins were precipitated by using the kit *PlusOne 2D Cleanup* (GE Healthcare, Uppsala, Sweden), as recommended by the manufacturer. The pellets were resuspended in rehydration buffer (8 M urea, 0.5% CHAPS, 10% glycerol, 0.5% IPG buffer pH 3–10, 7 mg/2.5 mL DTT, 0.002% bromophenol blue). Protein concentration was measured in each sample by Bradford protein assay [20]. After quantification, 1000 µg of liver proteins from each animal of the same group (n=6) were combined to constitute a pool that was submitted to proteomic analysis in triplicate, as described below.

### 2-DE Separation

Liver proteins (1000 µg) were taken from each pooled sample and mixed in a rehydration buffer to a volume of 400 µL and were then loaded onto 24-cm IPG strips (linear pH 3–10). Rehydration and first-dimensional IEF were performed on an IPGphor IEF system at 20°C with the following parameters: 50V for 12 h, 500V for 1 h, 1000V gradient for 1 h, then 10000V for a total of 40,000V. *Ettan DALTsix* (GE Healthcare, Uppsala, Sweden) with homemade 12.5% acrylamide gels was used for the second dimension of separation. Electrophoresis was performed at 15 mA/gel (80V) for 1 h and at 60 mA/gel (500V) until the bromophenol blue line reached the bottom of the gels. The resolved protein spots were stained with Colloidal Coomassie Brilliant Blue G-250 [21].

Gels were scanned with an Imagemaster scanner and all images were analyzed by using ImageMaster 2D Platinum 7.0 software from GE Healthcare (Uppsala, Sweden). Parameters used for spot detection were minimal area = 6 pixels, smooth factor = 2, and saliency = 300. The gel chosen as the reference in each group had the highest number of spots. The reference gel was then used for matching the corresponding protein spots between gels. Following the average mode of background subtraction, individual spot intensity volume was normalized with total intensity volume (summation of the intensity volumes obtained from all spots in the same 2-DE gel). The normalized intensity volume values of individual protein spots were then used to determine differential protein expression between the control and experimental groups. 2-D spots that exhibited a statistical significance were excised for future identification. Analysis of 2D-gel variability among the replicates of each experimental condition was taken by using the relative volume (% vol). The correlation coefficients among the triplicates were shown to vary from 0.87 to 0.96 (Figure S4).

### LC-MS/MS Analysis

Protein spots of interest were excised from the gel and destained three times with 25 mM ammonium bicarbonate (Ambic)/Acetonitrile (ACN) (50: 50 v/v) for 30 min. The destained gel pieces were dehydrated twice with ACN for 10 min and dried in a vacuum concentrator (Eppendorf, Hamburg, Germany). The dried gel pieces were rehydrated with 20 mM DTT in 50 mM Ambic for 40 min at 56°C. Excess reagent was removed and 55 mM iodoacetamide (IAA) in 50mM Ambic was added for 30 min RT. The remaining liquid was removed and washed with 25 mM Ambic, followed by dehydration with ACN. After their removal, the gels were dried again. For digestion, dried gels were incubated with 10 ng/µL trypsin in 25 mM Ambic for 15 min (Trypsin Gold Mass Spectrometry, Promega, Madison, USA). Peptides were sequentially extracted from the gels initially in 50% ACN (v/v) with 5% formic acid for 14 h at 37°C, then in 50% ACN (v/v) with 1% formic acid for 15 min, followed by 60% methanol (v/v) with 1% formic acid for 15 min and twice with 100% ACN at 45°C under sonication (40kHz/30W, Branson, Danbury, USA). Extracts were dried using a vacuum concentrator (Eppendorf, Hamburg, Germany) and kept at -20°C. Prior to MS identification, dried peptides were dissolved in 12 µL 0.1% formic acid. The peptides were identified and quantified by Liquid Chromatography Electrospray Ionization Quadrupole Time of Flight Mass Spectrometry (LC-ESI-Q-TOF MS) (Waters, Mildord, USA). MassLynx 4.1 SCN662 software (Waters, Mildord, USA) was used to submit the combined MS and MS/MS data to the MASCOT database search engine (http://www.matrixscience.com) (version 2007.12.04) based on International Protein Index (IPI) protein database restricted to taxonomies *Rattus* (Rat). The search was limited with a mass tolerance of 100 ppm and only one missed cleavage per peptide was allowed. For modification of peptides, cysteine carbamidomethylation (fixed) and methionine oxidation (variable) were considered. Significant matching proteins required score of >60. Accuracy between the theoretical and experimental obtained mass and pI were also considered. When two or more proteins with high scores were identified in the same spot, they were excluded from the analysis. Identified proteins were classified into five different categories according to their primary function [22].

### Immunoblot analysis

Western blots were performed as previously described [23], and protein extracts from livers were obtained by lysing homoneized tissue in a RIPA buffer (0.5% sodium deoxycholate, 0.1% SDS, 1% NP-40) supplemented with protease inhibitors (Roche Diagnostics, Mannheim, Germany). Protein samples (40 µg) were resolved on 10% or 12% Tris-HCl polyacrylamide gels and subsequently transferred to a PVDF membrane. Membranes were probed with commercially available rabbit polyclonal anti-ApoE (1:500 dilution), anti-GRP-78 (1:250) (Abcam, Cambridge, MA, USA), anti-β-tubulin (1:200) (Santa Cruz, CA, USA), and anti-GAPDH (1:5000) antibodies (Cell signaling), followed by HRP-conjugated anti-rabbit antibody (1:10000) and ECL Plus detection reagents (GE Biosciences, Piscataway, NJ). Relative ApoE and GRP-78 band densities were determined by densitometric analysis using the ImageJ software from National Institutes of Health. In all instances, density values of bands were corrected by subtraction of the background values. The results were expressed as the ratio of ApoE to that of β-tubulin and of GRP-78 to that of GAPDH.

### Statistical analysis

Fluoride concentration and lipid droplets’ data were analyzed using ANOVA and Tukey’s test for individual comparisons. Graph Pad InStat version 3.0 for Windows software (San Diego, CA, USA) was used and the significance level was set at 5%.

For proteomic data, statistical analysis was performed using ANOVA (p<0.05) available at ImageMaster 2D Platinum 6.0 software (GE Healthcare, Uppsala, Sweden). Only proteins with significantly altered levels were excised for identification by MS.

## Results

### F Analysis

Mean (±SD) serum F levels for control, 5 mg/L, and 50 mg/L F groups were 0.019 (± 0.001), 0.026 (± 0.007) and 0.072 (± 0.013) mg/L, respectively. All groups significantly differed from each other (p<0.05). Mean (±SD) liver F levels for control, 5 mg/L and 50 mg/L F groups were 0.042 (± 0.016), 0.034 (± 0.021), and 0.059 (± 0.006) mg/kg, respectively. F levels observed in the 50 mg/L F group were significantly higher when compared with the control and 5 mg/L F groups (p<0.05).

### Hepatic Histopathological Examinations

**Figure 1:**
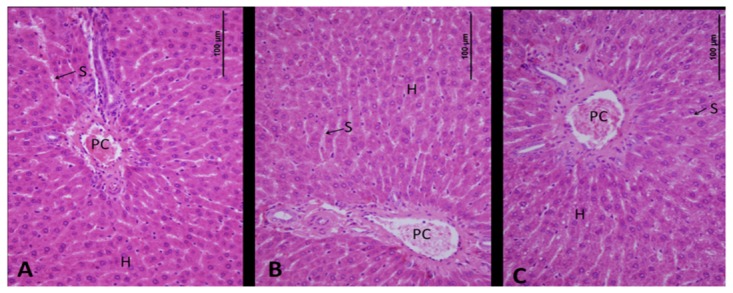
Photomicrographs of liver of rats treated chronically with drinking water containing different F concentrations. Overview of portal canal (PC), sinusoids (S), and hepatocytes (H), indicating normal morphology. (A) Control group, (B) group treated with 5 mg/L F and (C) group treated with 50 mg/L F.

**Figure 2:**
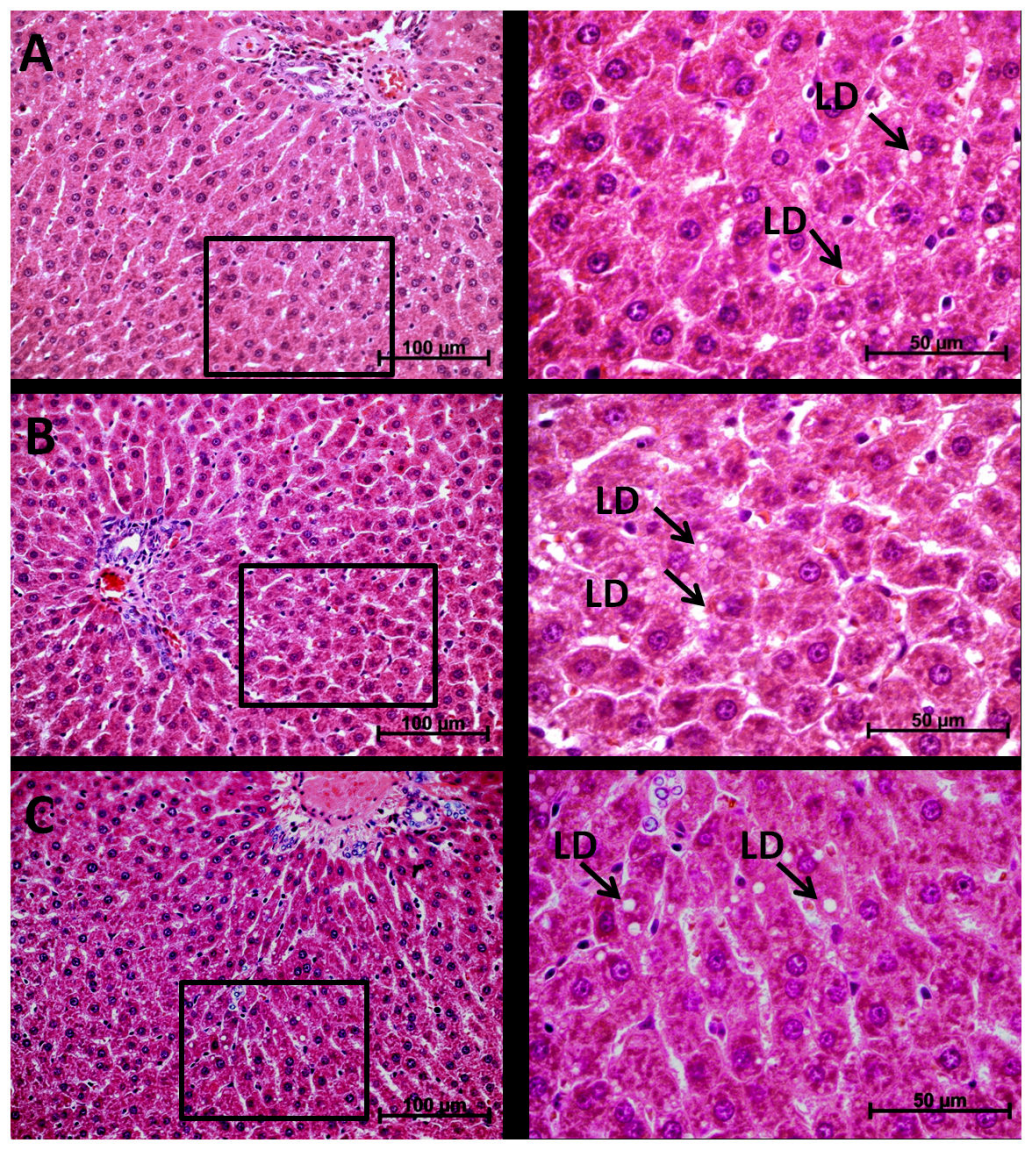
Photomicrographs of liver of rats showing macrovesicular lipid droplets (LD) in all groups. (A) control, (B) 5 mg/L F and (C) 50 mg/L F. In the left images, the regions bounded by the square are shown in greater increase in right images.

Morphometric analysis did not reveal alterations in the cellular structures (Figure 1). However, lipid droplets were found in all groups (Figure 2) and their existence was confirmed by osmium tetroxide staining (results not shown). The percentages were 22.8, 22.9, and 15.6% for control, 5 mg/L, and 50 mg/L F groups, respectively. Despite less lipid droplets being detected in the group treated with 50 mg/L F, the differences among the groups were not significant (p>0.05).

### Identification of Differentially Expressed and Unique Proteins

Gels from livers of the control, 5 mg/L, and 50 mg/L F groups presented 440, 401, and 405 total spots, respectively. Quantitative and qualitative intensity analysis is shown in Table S1. Representative 2D map of each comparison is also shown in the Supplementary Information (Figures S1-S3). In the comparisons between control *vs.* 5 mg/L F, control vs. 50 mg/L F, and 5 mg/L F vs. 50 mg/L F, 33, 44, and 29 proteins were differentially expressed, respectively, of which ~72% were successfully identified. Among them, 10 spots were up-modulated, while 9 were down-regulated in control livers when compared to 5 mg/L F treated rats. For the comparison between control *vs.* 50 mg/L F treated rats, 8 proteins were increased and 22 diminished in the latter group. Regarding the comparison between the groups treated with 5 mg/L F *vs.* 50 mg/L F, 5 proteins were significantly up-regulated and 8 down-modulated in livers of 50 mg/L F treated rats when compared with 5 mg/L F-treated rats.

**Figure 3:**
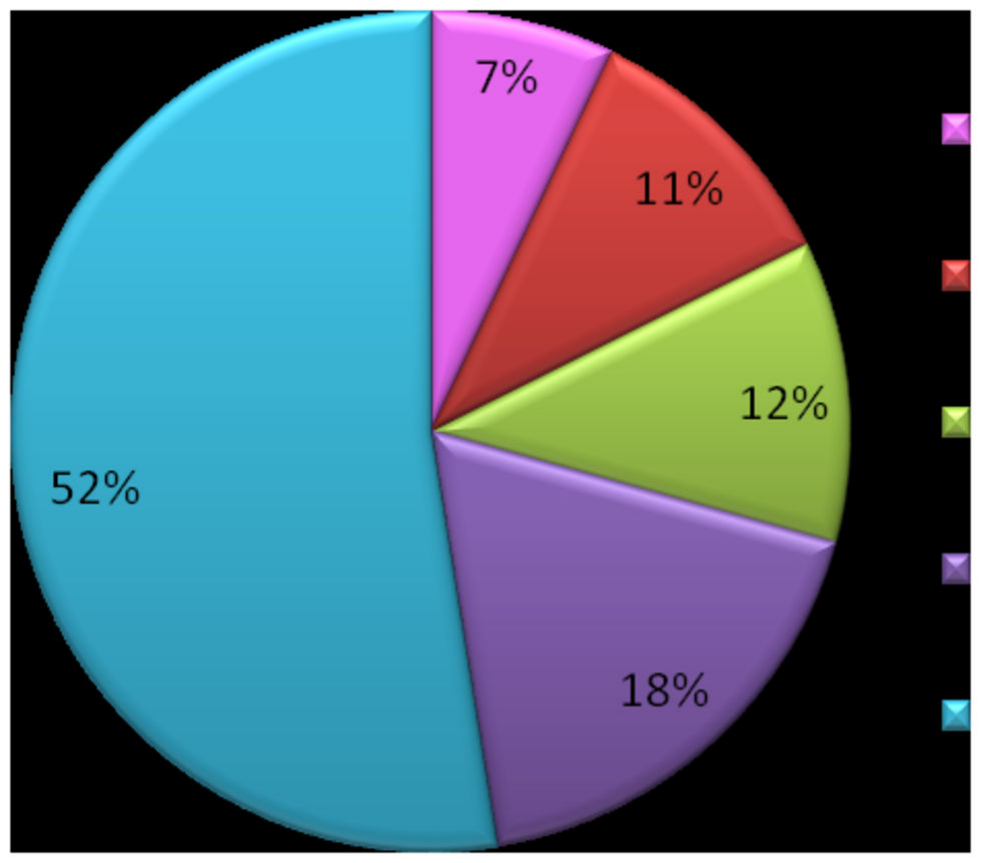
Functional distribution of proteins identified with differential expression in the liver of rats chronically treated with F or not. Category of protein based on its primary biological function according to Rison et al. (2000).

The treatment with 5 mg/L F and 50 mg/L F promoted the exhibition of 21 and 26 exclusively proteins, while 3 and 7 were shown to be excluded when compared to control rats. Identified proteins were divided into five functional categories, as follows: (1) metabolism, (2) information pathways, (3) processes, (4) transport, and (5) structure and organization of structure [22]. Percentages of each functional category from all identified proteins are represented in Figure 3.

### Validation of ApoE and GRP-78 proteins levels

**Figure 4:**
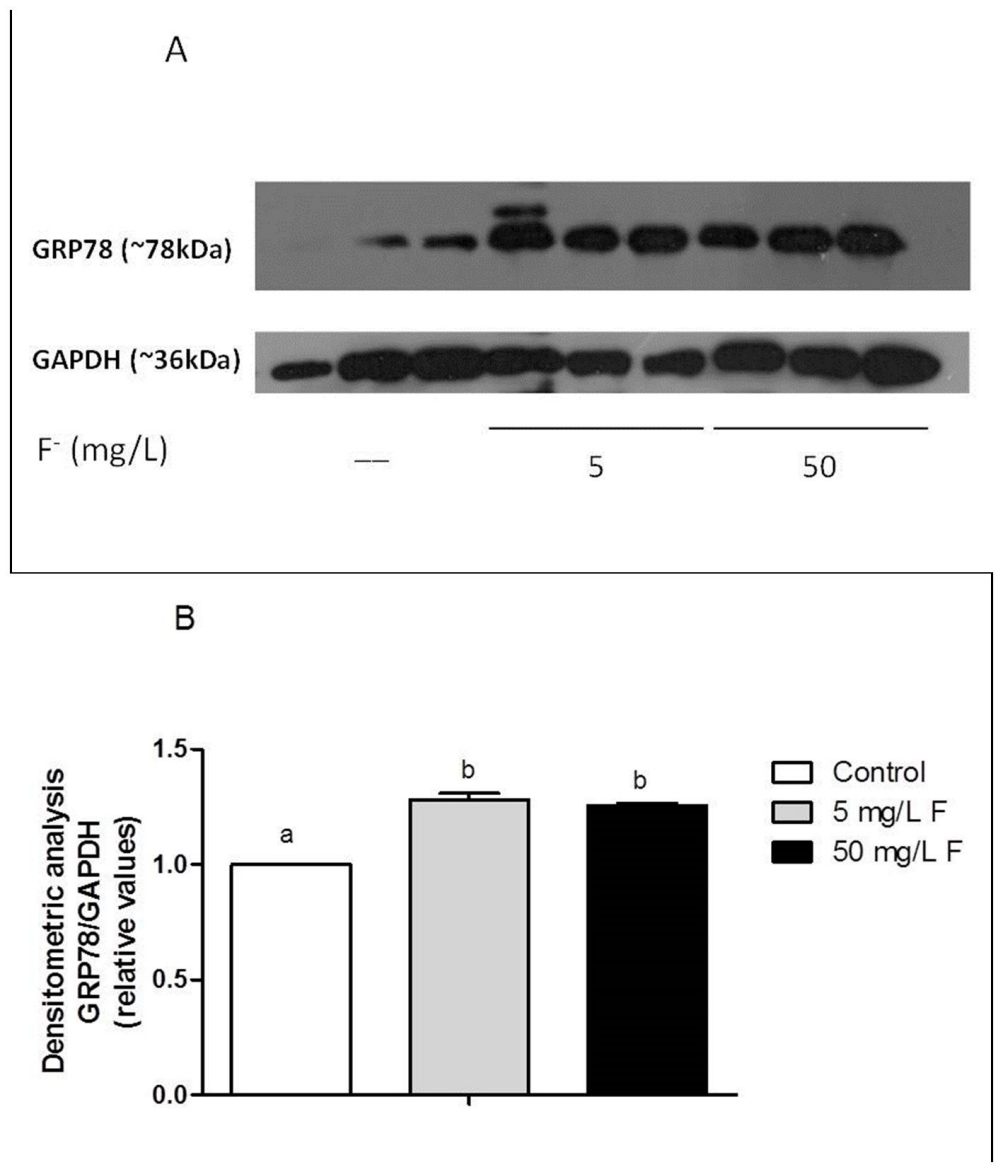
Effect of chronic treatment with F on the expression of protein GRP78 in the liver of rats chronically treated with F or not. (A) Representative blotting of GRP78 (~78 kDa) and constitutive GAPDH (~36 kDa) in samples of individual animals from each group (n=3). Samples are identified as indicated. Gels were performed in duplicate, each containing 3 different samples per respective group. (B) Densitometric analysis was done using imageJ software. The average of arbitrary values obtained for control group (n=6) was considered 1 and the averages of the other groups (n=6) were calculated in respect to control. Distinct letters indicate statistical significance (p<0.05).

**Figure 5:**
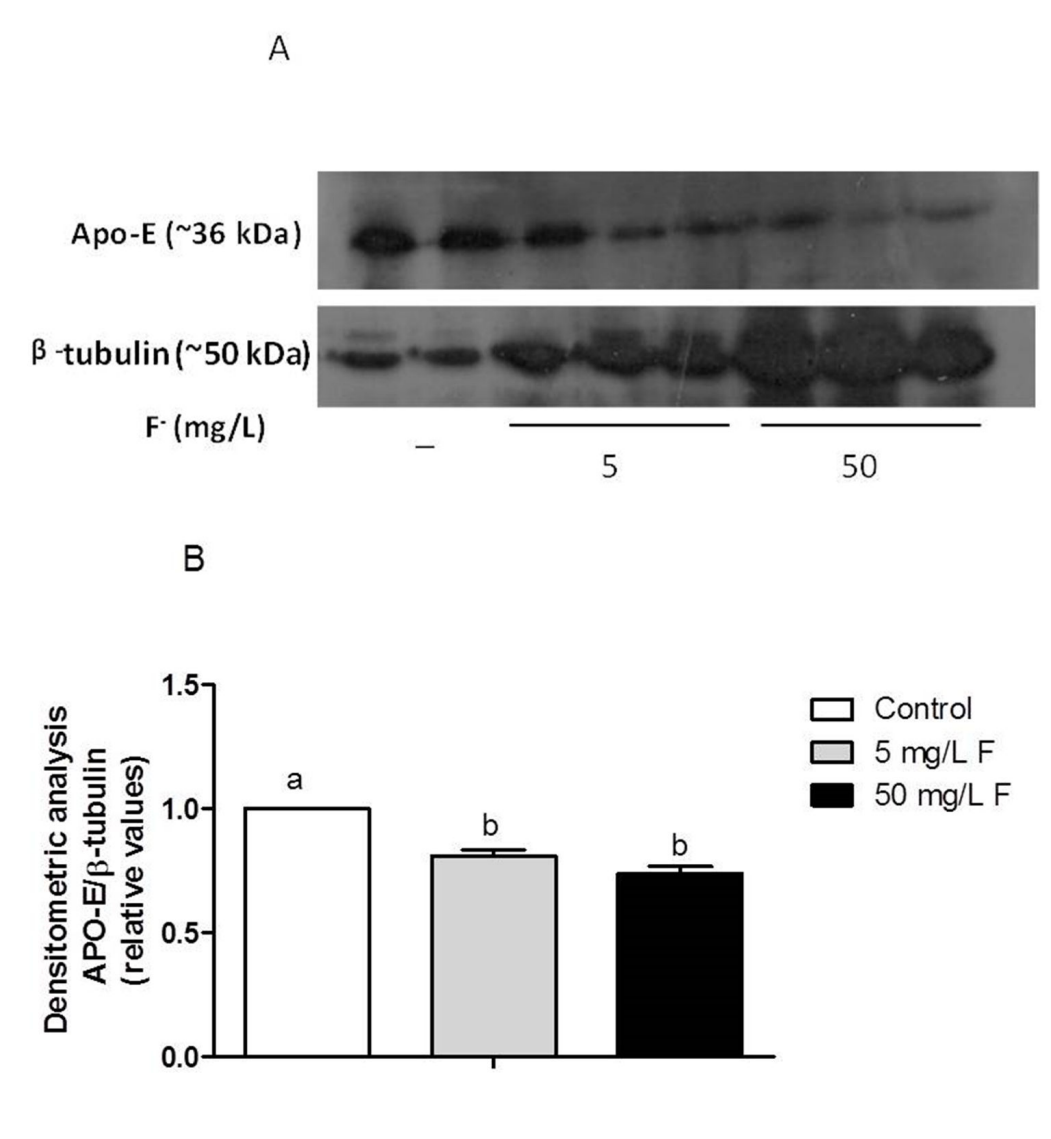
Effect of chronic treatment with F on the expression of protein Apo-E in the liver of rats chronically treated with F or not. (A) Representative blotting of Apo-E (~36 kDa) and constitutive β-tubulin (~50 kDa) in samples of individual animals from each group (n=2 or 3). Samples are identified as indicated. Gels were performed in duplicate, each containing 3 different samples per respective group. (B) Densitometric analysis was done using mage J software. The average of arbitrary values obtained for control group (n=6) was considered 1 and the averages of the other groups (n=6) were calculated in respect to control. Distinct letters indicate statistical significance (p<0.05).

Apolipoprotein E (ApoE) and 78-kDa glucose-regulated protein (GRP-78) proteins present lipid transport and anti-oxidative stress functions, respectively [24]. Because they were shown to be modulated by F as assessed by the proteome and may be involved in the alterations in lipid droplets in liver of F-treated rats, we selected them for validation of their quantitative expression. Although GRP78 and Apo-E were shown to be exclusively present and absent respectively, in F-treated groups compared to control, western blotting analysis showed that GRP-78 protein expression in liver was up-regulated, while Apo-E expression was down-modulated in liver in response to F (Figures 4 and 5).

## Discussion

Proteomic alterations that happen in kidneys following F intoxication are relatively well described in the literature [25,26], but information regarding other organs and, especially liver, is scarce. Knowledge of the molecular mechanisms underlying liver F toxicity is very important because this organ has an important role in detoxification of the organism. In the present study, we have shown that F alters the expression and diversity of many hepatic proteins. The hepatotoxicity caused by 50 mg/L F was followed by the enhancement of protein arginase-liver type (ARG) expression. This protein is an important marker of hepatocellular injury. It was reported that ARG is highly sensitive to liver injury, compared to other liver-type ornithine carbamoyltransferase (OCT) and argininosuccinate synthase (AS) and two conventional hepatic markers, aspartate and alanine aminotransferases [27]. Alterations in 8 proteins related to structure and organization of structure were observed in the treated groups when compared with control (Table S1). This is consistent with observations that F can alter the cytoskeleton and affect cell mobility [3].

In most of the cases, treatment with F resulted in an increase of expression or lack of expression of proteins related to processes. Perodoxiredoxin (PRDX) comprises a family of proteins with an important antioxidant role [28,29]. They are differentially dispersed within cells and can protect against different stresses [28,29]. The absence of PRDX 1 (mitochondrial) and -5 (cytoplasmic) in the group treated with 50 mg/L F can be associated with F-induced oxidative stress [3,7,30].

It has been suggested that F can bind to a subunit of guanine nucleotide-binding protein (G protein) activating this protein by changing its conformation [4]. Ge et al. [31] observed down-regulation of the G protein in the brains of rats exposed to high F concentration (100 mg/L F) in attempt to avoid overstimulation by this ion. In line with this, in the present study, it was observed the absence of the beta subunit of this protein in the experimental groups. A similar finding was the lower expression of phosphatidylethanolamine binding protein 1 (PEBP-1) in the group treated with 50 mg/L F. This protein is also involved in the G-protein mediated-signaling pathways [32].

Exposure to F altered the expression of different proteins associated with proteosomes. Proteosome subunit alpha type-3 was increased upon exposure to F, which was also observed in the brain of rats treated with high F dose [31]. It was suggested that this increase could lead to an increment of protein degradation possibly through decomposition of normal proteins without recognition by ubiquitin. Also, the protein ubiquitin carrier was absent in this group, corroborating the hypothesis that F may increase protein degradation. Instead, other isoforms such as the Proteosome subunit beta type-4 and Proteosome subunit alpha type 5 were diminished or absent, respectively, in the group treated with 50 mg/L F, suggesting reduction of protein degradation after F. These data do not allow us to affirm whether or not F enhances or decreases protein degradation, but this ion certainly promotes dysfunction of protein degradation, since it modulates the expression of key protein involved in this process.

Proteins related to protein synthesis, as is the case for elongation factors (EF), promote the GTP-dependent binding of a tRNA-aminoacyl to the ribosome [33]. Besides participating in the translation, EF-1-α-1 forms complex with other cell components, such as tubulin and actin [34,35] and acts in cell process, including embryogenesis, senescence, oncogenic transformation, proliferation, and organization of the cell cytoskeleton [36]. Recently, it was demonstrated the involvement of EF-1-α-1 in apoptotic events induced by oxidative stress [37,38]. In the present study, it was observed an increase in the expression of EF-1-α-1 and EF-2 in the group treated with 50 mg/L F, which could contribute to an increase in apoptotic events that have shown to be induced by F [3].

**Figure 6:**
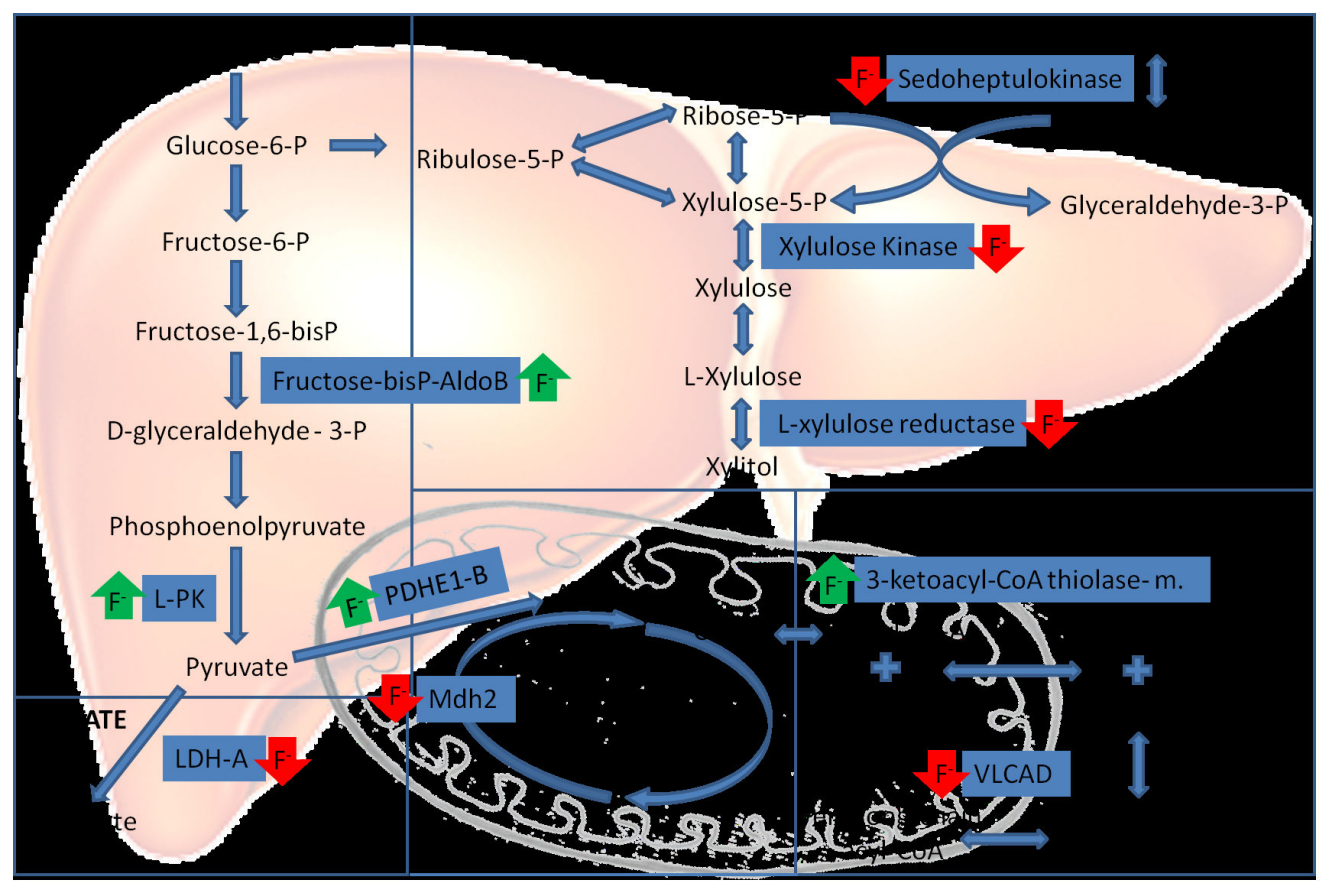
Simplified scheme of F-induced alterations in the expression of proteins related to energetic metabolism pathways in liver. F^-^ (fluoride ion); red arrows indicate increased expression and green arrows indicate decreased expression; P (phosphate); Fructose-bisP-AldoB (Fructose-bisphosphate aldolase B); L-PK (R-type isoform of piruvate kinase isozymes R/L); PDHE1-B (Pyruvate dehydrogenase E1 component subunit beta, mitochondrial); LDH-A (L-lactate dehydrogenase A chain); Mdh2 (Malate dehydrogenase); OAA (oxaloacetate); VLCAD (Very long-chain specific acyl-CoA dehydrogenase, mitochondrial); 3-Ketoacyl-CoA thiolase – m.(3-Ketoacyl-CoA thiolase, mitochondrial).

Forty-nine proteins associated to metabolism were identified with altered expression in the present study (Table S1). Among them, nine are related to carbohydrate metabolism. F has been shown to inhibit the activity of enzymes involved in the citric acid cycle [3]. The absence of malate dehydrogenase in the group treated with 50 mg/L F might contribute to the reduction of the flux of the citric acid cycle in the presence of high doses of this ion. Xiong found a dose-response relationship between the levels of F in the drinking water and damage in the hepatic function in children. The authors observed an increase in the activity of LDH in the serum of children ingesting water containing more than 2 mg/L F. Ge [30] also observed an overexpression of this enzyme in the brain of rats treated with F. Instead the L-lactate dehydrogenase (LDH) A chain, related to lactate pathway and sedoheptulokinase, involved in the pentoses cycle, were absent in F-treated groups, indicating that in presence of F these pathways suffer alteration. This might be related with a compensatory overexpression of enzymes involved in the glycolytic pathway such as fructose-bisphosphate aldolase B, pyruvate dehydrogenase E1 component subunit beta, mitochondrial and isoform R-type of piruvate kinase isozymes R/L. An increase of fructose-bisphophate aldolase B in kidneys of rats that received 5 mg/L was reported previously [26], which could be an initial signal of alteration in cell metabolism after low F doses. Since F is able to inhibit enolase, the overexpression of aldolase (an upstream glycolytic enzyme) could to be an attempt of cells to keep the efficiency of the glycolytic pathway [26,39]. In the present study, many enzymes related to the glycolytic pathway had their expression increased in the experimental groups (Table S1), indicating that F intensified this pathway and at the same time decreased the alternative lactate and pentoses phosphate pathways (Figure 6).

Among the altered metabolic proteins, nine of them were related to lipid metabolism (Table S1). The group treated with 50 mg/L F had alterations in proteins involved in the beta oxidation of fatty acids. The beta-oxidation is a multi-step process by which fatty acids are broken down to produce energy. It occurs in mitochondria and peroxisomes by mechanisms involving dehydrogenation, hydration, redehydrogenation, and thiolytic cleavage [40]. In mitochondria, the first oxidation step of long-chain fatty acids is catalyzed by the very long-chain acyl-CoA dehydrogenase (VLCAD) [41] and the fourth oxidation step is catalyzed by 3-Ketoacyl-CoA thiolase, mitochondrial [42]. The first protein was absent while the second was increased in the group treated with 50 mg/L F, suggesting that the catalyzation of long-chain fatty acids may be compromised while the fatty acid beta-oxidation may be stimulated (Figure 6). Increases in enzymes related to fatty acids beta oxidation in the presence of high F has been reported previously in kidneys [25,26], suggesting a vigorous state of fatty acids metabolism. Differently to what was observed for mitochondrial enzymes, we observed an increase in the expression of proteins involved in the first step, such as isoform 1 Peroxisomal acyl-coenzy A oxidase 1 (ACOX1) and Peroxisomal trans-2-enoyl-CoA reductase (PX-2,4-DCR1). ACOX1 catalyzes the oxidation of long and medium straight chain substrates [43] and PX-2,4-DCR1 is an additional enzyme required for mono- and polyunsaturated fatty acids [44]. Also, F abrogated the expression of proteins related to the fourth step (peroxisomal acetyl-Coenzyme A acyltransferase 1A (Acaa1a) and 3-Ketoacyl-CoA thiolase B), involved in the peroxisomal beta oxidation of straight-chain fatty acids [40]. We conclude that F alters expression of several phase-dependent mitochondrial and peroxisomal proteins involved in the fatty acid metabolism, promoting its enhancement. The absence of VLCAD after F does not rule out the possibility of up-regulation of F-induced fatty acid beta oxidation. Instead, it may be a consequence of a compensatory mechanism induced by an enhancement of the first step in peroxisomes, since many of them can be beta-oxidized in both mitochondria and peroxisomes [43].

A great number of proteins associated with amino acids metabolism were found with alterations in the present study. Among them, three proteins that are members of the glutathione S-Transferase (GST) family were identified with increased expression in the presence of F. They are related to the degradation of amino acids and also to xenobiotics metabolism [45,46]. An increase in the activity of GST in the liver of rats treated with F was associated with an attempt to reduce the oxidative stress caused by F [46], which is in line with the present study.

Regarding energetic metabolism, five proteins were found with altered expression. ATP-synthase mitochondrial is a type F ATPase located in the inner mitochondrial membrane that catalyses the formation of ATP from ADP and P_i_. It is composed of two distinct components: F_1_ (peripheral membrane protein that faces the mitochondrial matrix) and F_0_ (transmembrane). F_1_ is a complex of nine different subunits from five different types (α_3_β_3_γδε), while F_0_ is composed of three subunits (a, b, and c) in a proportion of ab _2_c_10-12_ [42]. In the present study, we identified ATP synthase subunit alpha, mitochondrial with decreased expression in the 5 mg/L F group in comparison to control, ATP synthase subunit beta, mitochondrial that was absent in the experimental groups, and ATP synthase gamma chain that was absent in the 50 mg/L F group. The lower expression of these proteins in the presence of F could cause a decrease in the production of ATP, which has been consistently reported to occur in the presence of F [3,4]. We also identified Cytochrome b-c1 complex subunit 1, mitochondrial with increased expression in a dose-response manner in the presence of F and Cytochrome c oxidase subunit 5A, mitochondrial with lower expression in the 50 mg/L F in comparison to 5 mg/L F group. Both of them are soluble proteins located in the intermembrane space of mitochondria that take part in the complexes III and IV of the electron transport chain, respectively [42]. It has been reported that NaF induces an increase in the level of cytochrome c released from mitochondria into the cytosol in a dose-dependent manner in human gingival fibroblasts, with implication in the induction of apoptosis [47]. Thus, the reduction of cytochrome c oxidase seen in the group that received 50 mg/L F could be an attempt to diminish the apoptotic effect induced by F. In addition, exposure to F reduces the production of ATP [3]. Thus, the dose-dependent overexpression of cytochrome b-c1, “upstream” to cytochrome c, could be an attempt of cell to regulate the energetic flow.

As for proteins related to information pathways, two chaperones, namely “T complex protein 1 subunit gama” and “Heme binding protein 1, isoform CRA-a” were found to be absent in the group treated with 5 mg/L F. This finding could indicate difficulty in protein folding [42] in the presence of low F dose. On the other hand, another chaperone (Hsp60) was increased in the group treated with 50 mg/L F compared to control. Xu et al. [48] also observed an increase in the expression of a protein from this family (Hsp70) in culture of osteoblasts exposed to F, suggesting increased oxidative folding in the present of high doses of this ion. It has been reported that Hps 70 is rapidly activated during the process of cellular response to environmental stress, suggesting that this protein may mediate chronic intoxication by F [3]. GRP-78 is involved with the assembly of protein complexes inside the endoplasmic reticulum. Its expression is induced by physiological stresses that affect protein glycosylation [49]. In the present study, proteomic analysis revealed that this protein was present only in the experimental groups. However, western blotting analysis showed an increase of this protein in the groups treated with F, when compared to control. This protein was also shown to be increased in culture of brain tumor cells treated with F (95 mg/L NaF for 2 h) [50]. The increase of GRP78 and Hsp60 in the presence of F suggests endoplasmic reticulum (ER) stress. ER stress occurs when nascent proteins are not folded properly or are misfolded, leading to the initiation of the unfolded protein response, as the unfolded proteins accumulate in the ER [51]. Kubota [51] demonstrated that NaF induces an ER stress response in the ameloblast derived LS8 cell line.

Regarding proteins related to transport, it was observed that Annexin A presented a lower expression in the 50 mg/L F group when compared with the 5 mg/L F group. This could indicate an intracellular retention of calcium described in some tissues in cases of exposure to F [3]. The Fatty acid-binding protein, liver (L-FABP) had a lower expression in the group treated with 5 mg/L F compared with the control, indicating a reduction in the combat of oxidative stress [52,53] The most remarkable proteomic finding in this category was the absence of Apo-E in the experimental groups. The Western blotting analysis revealed that in fact this protein was down-regulated in the experimental groups. Deficiency in this protein is related to induction of oxidative stress [24].

Related to this, the histological analysis revealed the presence of lipid droplets in all groups. One possible reason for this finding was the administration of AIN-93M diet. In a recent publication, it was shown that this diet leads to accumulation of hepatic fat, due to its higher carbohydrate and calorie content [16]. However, animals treated with high concentration F presented a tendency of reduction in the percentage of lipid droplets, despite the difference among the groups was not significant. The lack of significant differences might be due to the period when samples were collected (80 days of life). It has been shown that from day 30 until day 90 of NaF administration, the morphological changes show a gradual decrease in intensity, which could be a result of adaptive mechanisms of the organism to F [15]. The tendency for reduction of lipid droplets might be due to the treatment with F, since western blotting analysis confirmed the down-regulation of Apo-E and up-regulation of GRP78 upon treatment with F. The increased expression of GRP78 inhibits ER stress, reducing hepatic steatosis in mice [54]. In addition, it was recently reported that ER stress-dependent hepatic steatosis was diminished in the livers of the VLDLR-deficient and ApoE-deficient mice compared to the wild-type mice, suggesting that steatosis can be reduced when the availability of lipoproteins to deliver fat to liver, allowing intracellular triglyceride accumulation, is diminished [55].

Briefly, exposure to F altered the expression of liver proteins belonging to all the functional categories, especially those related to metabolism. More pronounced alterations were seen for the 50 mg/L group. Proteins that were not detected upon exposure to F or that had their expression induced in the presence of F are potential biomarkers of toxicity and should be better investigated. Since this is the first study involving proteomic analysis of livers in rats exposed to different doses of F, these findings indicate important pathways and cellular processes affected by exposure to this element that should be addressed in more detail in future studies.

## Supporting Information

Figure S1 **2D gel analysis of rat liver proteome between control and low level of F.** Selected spots in green and in red represent those with differential expression and exclusives, respectively, in the comparison between control (A) *vs* 5 mg/L F-treated rats (B). The linear pH and the molecular weights are indicated in boxes in the horizontal and vertical, respectively. Numbers in blue represent common spots indicated for references when matching gels.
(TIF)

Figure S2 **2D gel analysis of liver proteome between control and high level of F.** Selected spots in green and in red represent those with differential expression and exclusives, respectively, in the comparison between control (A) *vs* 50 mg/L F-treated rats (B). The linear pH and the molecular weights are indicated in boxes in the horizontal and vertical, respectively. Numbers in blue represent common spots indicated for references when matching gels.
(TIF)

Figure S3 **2D gel analysis of liver proteome between low and high levels of F.** Selected spots in green and in red represent those with differential expression and exclusives, respectively, in the comparison between 5 mg/L F (A) *vs* 50 mg/L F-treated rats (B). The linear pH and the molecular weights are indicated in boxes in the horizontal and vertical, respectively. Numbers in blue represent common spots indicated for references when matching gels.
(TIF)

Figure S4 **2D gel variability analysis.** Scatter plot of binary comparisons among the ratios of relative spots volumes detected in the representative gel (replicate 1) and the respective replicates (replicates 2 and 3). (A) Control; (B) 5 mg/L F-treated rats; (C) 50 mg/L F treated-rats.
(TIF)

Table S1 **Identified proteins with expression significantly altered in the liver of rats treated chronically with different doses of F.**
(XLSX)
