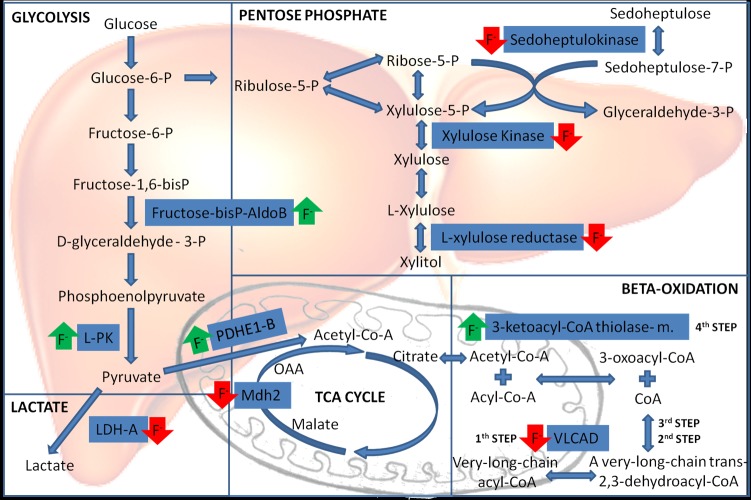# Correction: Proteomic Analysis of Liver in Rats Chronically Exposed to Fluoride

**DOI:** 10.1371/annotation/54b8fb45-bb22-469e-96b3-77e99ba62b77

**Published:** 2013-11-08

**Authors:** Heloísa Aparecida Barbosa da Silva Pereira, Aline de Lima Leite, Senda Charone, Janete Gualiume Vaz Madureira Lobo, Tania Mary Cestari, Camila Peres-Buzalaf, Marília Afonso Rabelo Buzalaf

Due to issues in the typesetting process, there were errors in Figures 3,4, and 6. Correct versions of these Figures are available below.

Figure 3: 

**Figure:**
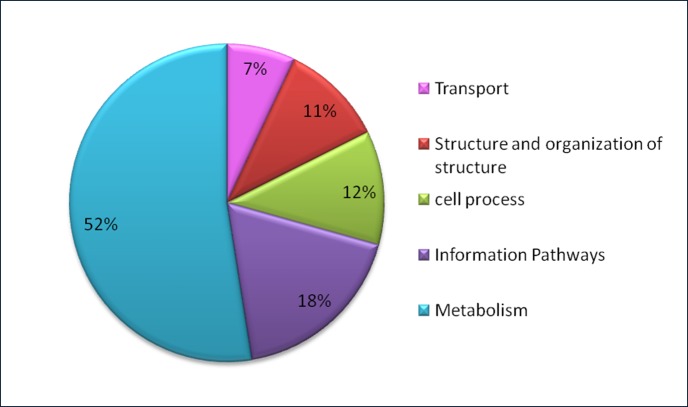


Figure 4: 

**Figure:**
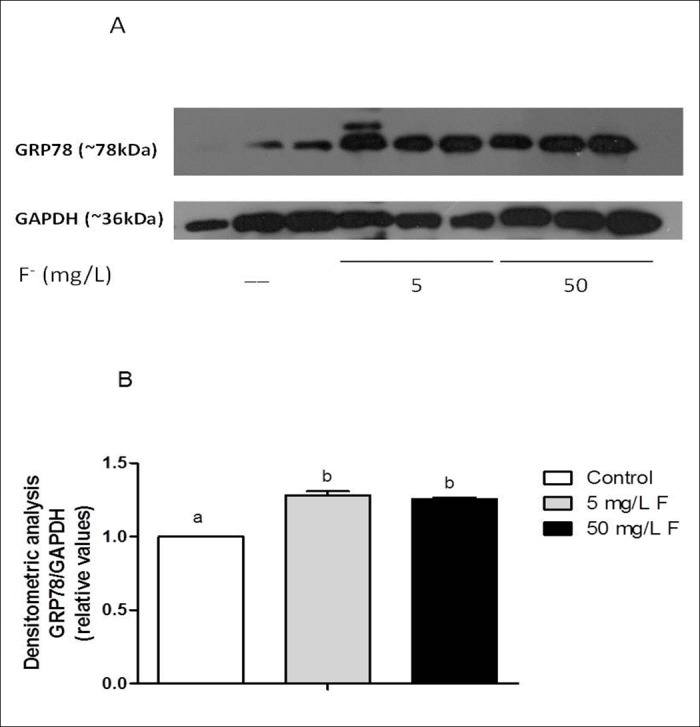


Figure 6: 

**Figure:**