# Supplementary figures and images for: Proteomic Analysis of Liver in Rats Chronically Exposed to Fluoride
Source: PLoS One. 2013 Sep 17;8(9):e75343. doi: 10.1371/journal.pone.0075343 (PMC3775814; doi:10.1371/journal.pone.0075343)

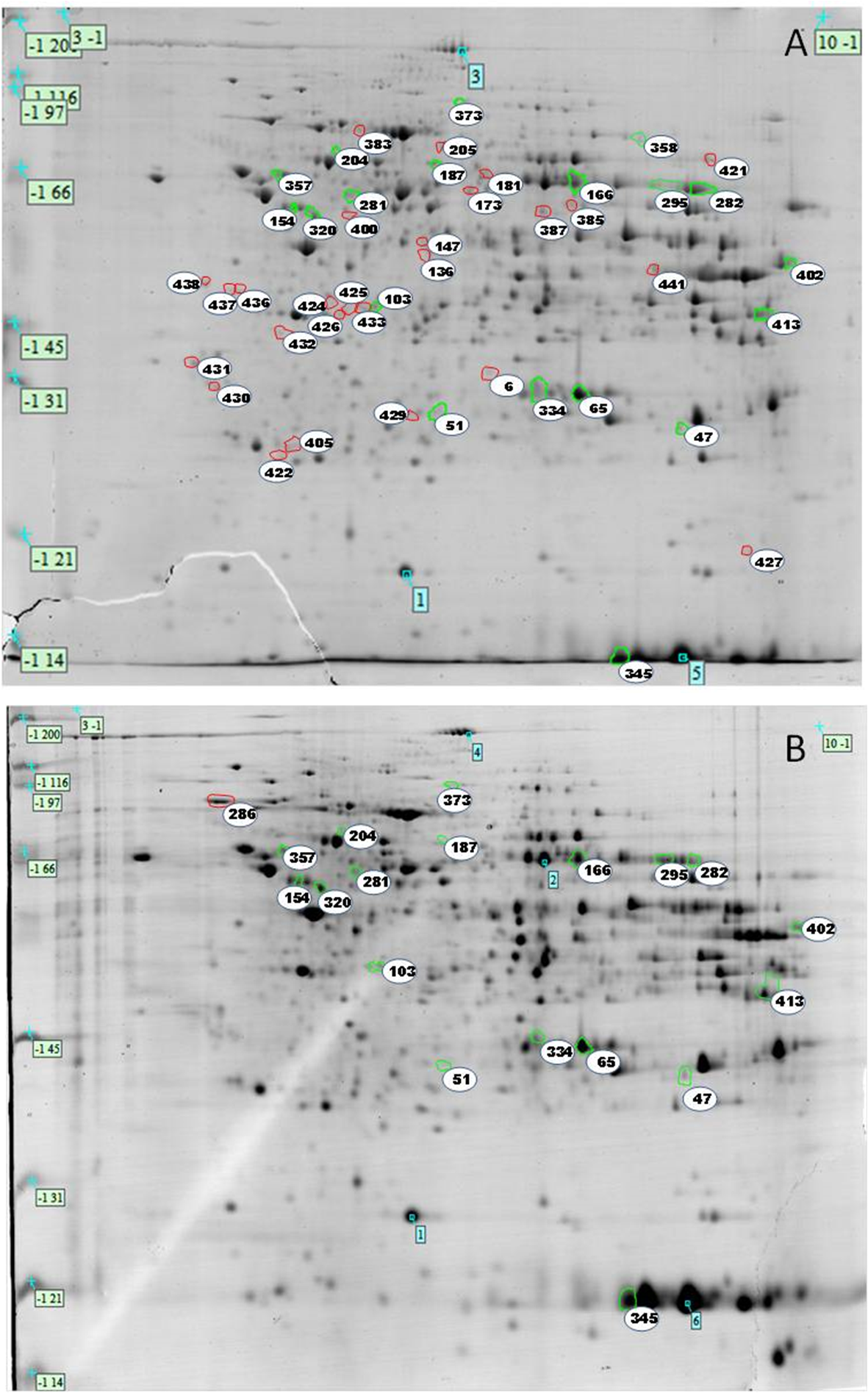

Supplement: Figure S1 — 2D gel analysis of rat liver proteome between control and low level of F. Selected spots in green and in red represent those with differential expression and exclusives, respectively, in the comparison between control (A) vs 5 mg/L F-treated rats (B). The linear pH and the molecular weights are indicated in boxes in the horizontal and vertical, respectively. Numbers in blue represent common spots indicated for references when matching gels. (TIF) [file pone.0075343.s001.tif]

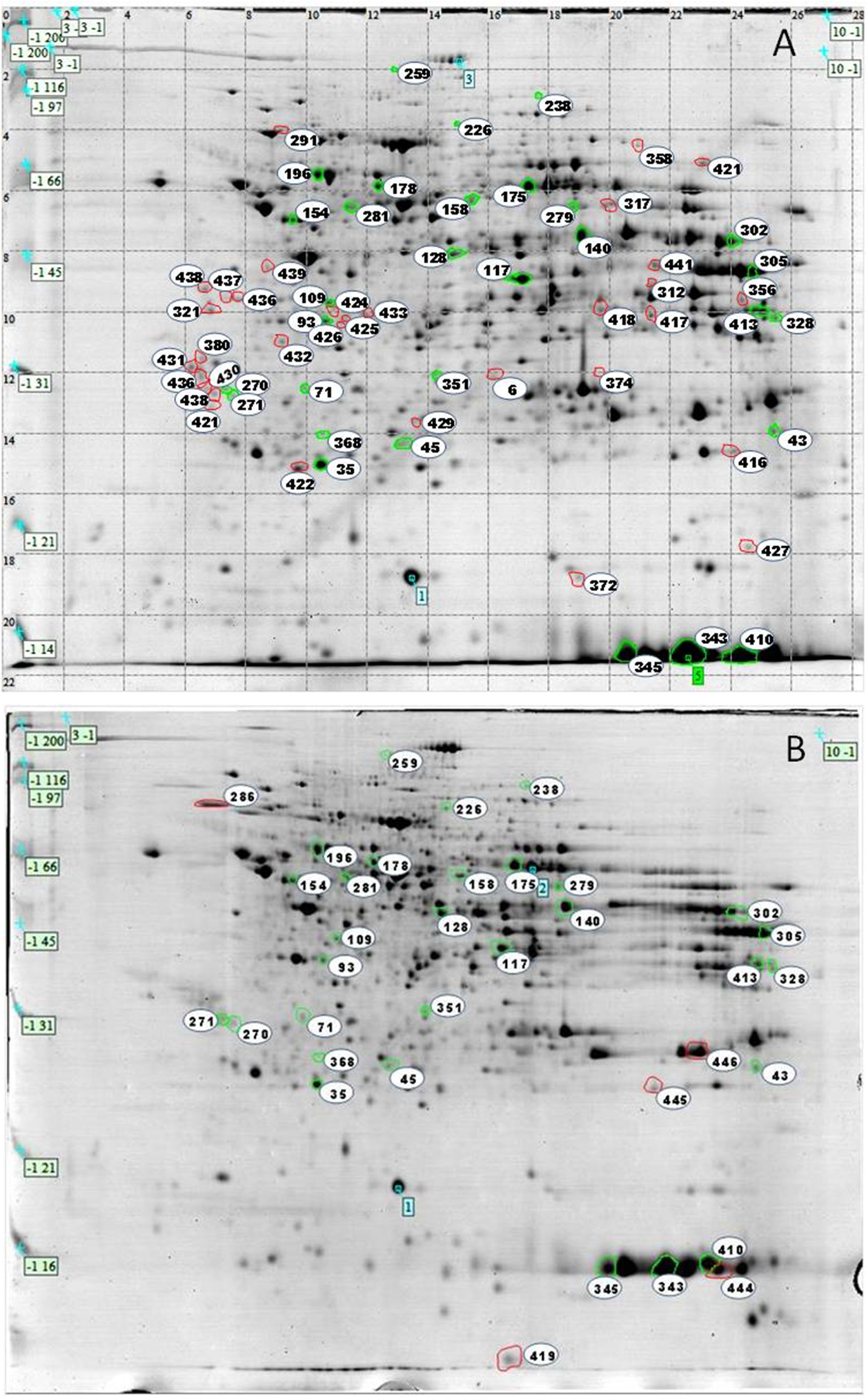

Supplement: Figure S2 — 2D gel analysis of liver proteome between control and high level of F. Selected spots in green and in red represent those with differential expression and exclusives, respectively, in the comparison between control (A) vs 50 mg/L F-treated rats (B). The linear pH and the molecular weights are indicated in boxes in the horizontal and vertical, respectively. Numbers in blue represent common spots indicated for references when matching gels. (TIF) [file pone.0075343.s002.tif]

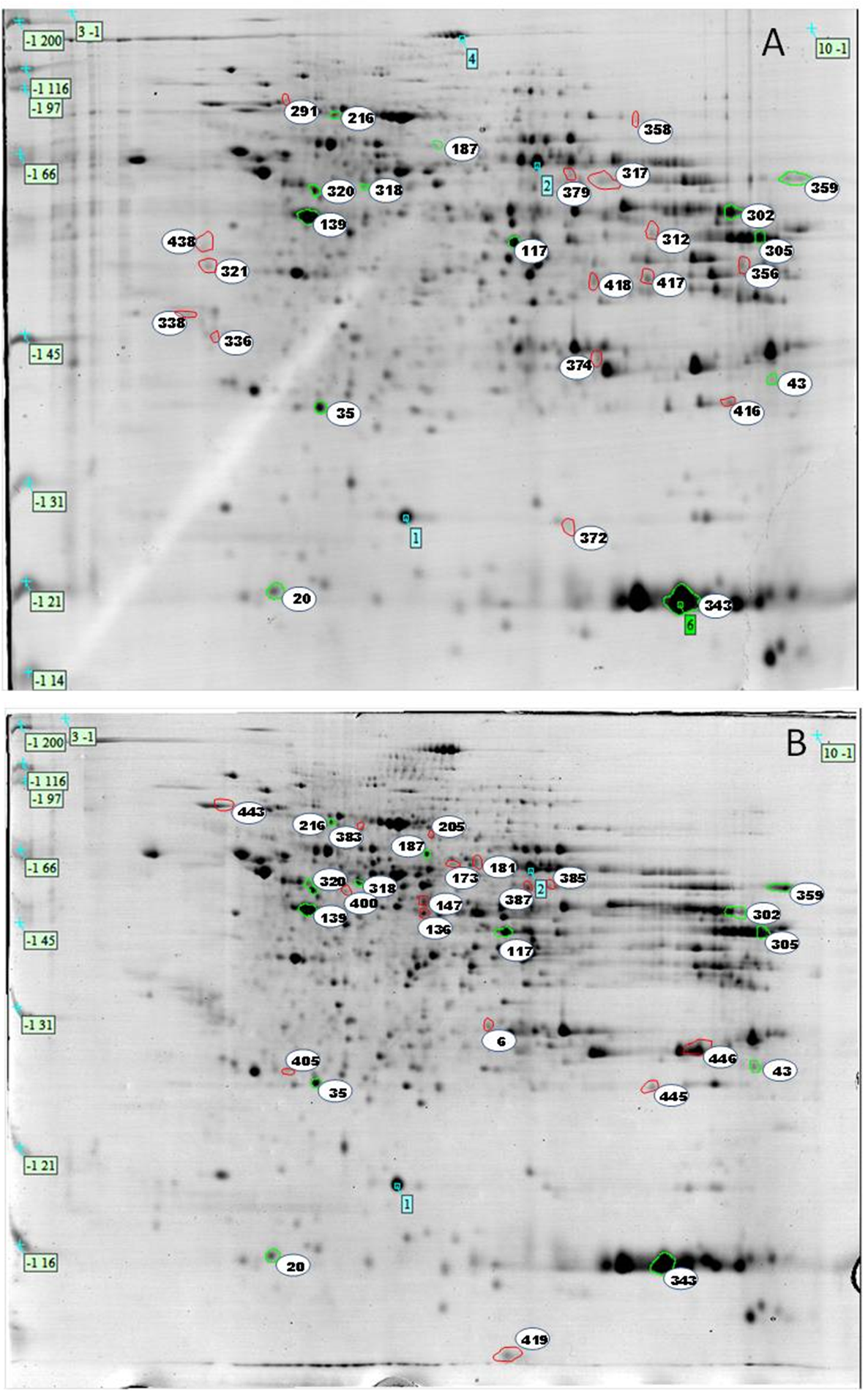

Supplement: Figure S3 — 2D gel analysis of liver proteome between low and high levels of F. Selected spots in green and in red represent those with differential expression and exclusives, respectively, in the comparison between 5 mg/L F (A) vs 50 mg/L F-treated rats (B). The linear pH and the molecular weights are indicated in boxes in the horizontal and vertical, respectively. Numbers in blue represent common spots indicated for references when matching gels. (TIF) [file pone.0075343.s003.tif]

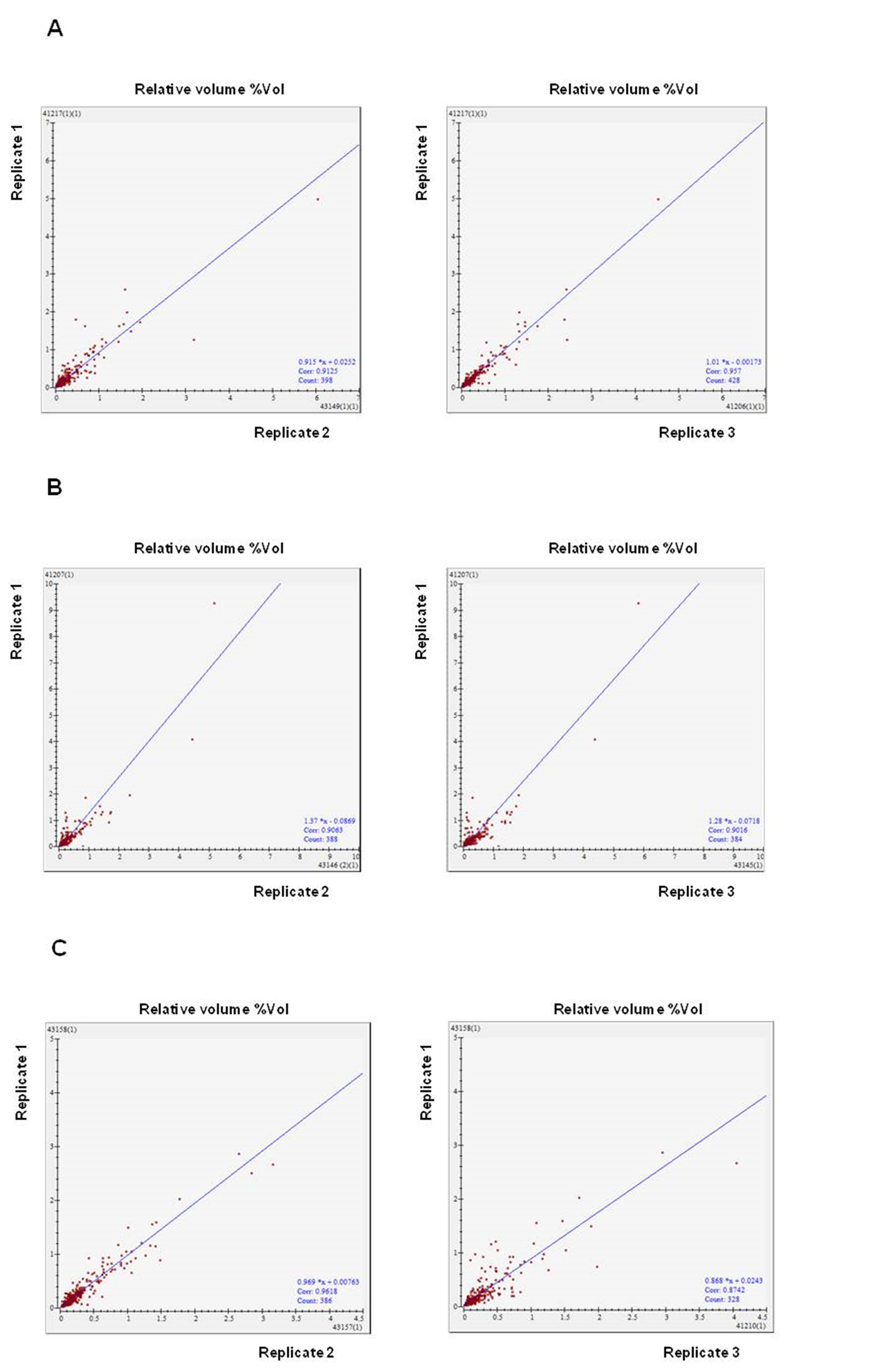

Supplement: Figure S4 — 2D gel variability analysis. Scatter plot of binary comparisons among the ratios of relative spots volumes detected in the representative gel (replicate 1) and the respective replicates (replicates 2 and 3). (A) Control; (B) 5 mg/L F-treated rats; (C) 50 mg/L F treated-rats. (TIF) [file pone.0075343.s004.tif]
